# Tough, stable and self-healing luminescent perovskite-polymer matrix applicable to all harsh aquatic environments

**DOI:** 10.1038/s41467-022-29084-z

**Published:** 2022-03-14

**Authors:** Yuncong Liu, Tao Chen, Zhekai Jin, Mengxue Li, Dongdong Zhang, Lian Duan, Zhiguo Zhao, Chao Wang

**Affiliations:** 1 Key Lab of Organic Optoelectronics & Molecular Engineering, Department of Chemistry, Tsinghua University, Beijing, 100084 PR China; 2 China Huaneng Clean Energy Research Institute, Beijing, 102218 PR China

**Keywords:** Supramolecular polymers, Polymers, Polymers

## Abstract

Gelatinous underwater invertebrates such as jellyfish have organs that are transparent, luminescent and self-healing, which allow the creatures to navigate, camouflage themselves and, indeed, survive in aquatic environments. Artificial luminescent materials that can mimic such functionality can be used to develop aquatic wearable/stretchable displays and water-resistant devices. Here, a luminescent composite that is simultaneously transparent, tough and can autonomously self-heal in both dry and wet conditions is reported. A tough, self-healable fluorine elastomer with dipole–dipole interactions is synthesized as the polymer matrix. It exhibits excellent compatibility with metal halide perovskite quantum dots. The composite possesses a toughness of 19 MJ m^−3^, maximum strain of 1300% and capability to autonomously self-heal underwater. Notably, the material can withstand extremely harsh aqueous conditions, such as highly salty, acidic (pH = 1) and basic (pH = 13) environment for more than several months with almost no decay in mechanical performance or optical properties.

## Abstract (web summary)

Fabrication of perovskite-polymer composites that simultaneously possesses strong mechanical strength, high stretchability, high stability towards harsh aqueous environments and self-healing properties is challenging. Here, the authors report a luminescent composite that is simultaneously transparent, tough and can autonomously self-heal in both dry and wet conditions.

## Introduction

Recently metal halide perovskites have become appealing materials for luminescent devices. They have obvious advantages of high quantum efficiency, widely tunable bandgap, narrow emission width and full width to half-maximum (FWHM)^1–5^. However, accompanied with excellent optoelectronic performance, the perovskite quantum dots (QDs) suffer from poor stability. Because of the low formation energy and highly mobile ionic structure with surface traps, perovskite nanoparticles are very unstable against external factors including moisture, temperature and chemical environment^6–10^. The poor stability greatly limits their practical application in optoelectronic devices. For wearable devices in marine applications, the constant mechanical deformations and salty water environment make the stability an even tougher issue.

Much effort has been devoted to stabilize the perovskite QDs^11–17^. Embedding them into polymer matrix is an effective route^18–21^. The long polymer chains can form compact encapsulation around the perovskite QDs to protect them from contacting with the environment. Polymethyl methacrylate (PMMA) and polystyrene (PS) are widely used as the polymer matrix but they are not stretchable^22–24^. Recently, He et al. used polyacrylate organogels as matrix to make stretchable luminescent gels that are stable in water but the mechanical strength is relatively low^25^. For practical applications such as wearable optoelectronics, strong mechanical strength, high stretchability and optoelectronic stability against all types of harsh environments should be satisfied^26,27^. In addition, the ability to recover its mechanical and optoelectronic performance, just like the jellyfish does, is also highly important considering the constant deformations and physical damages in the application of wearable electronics^28,29^. However, up to now, a perovskite-polymer material that simultaneously possesses strong mechanical strength, high stretchability, high stability towards all harsh aqueous environments and self-healing property has not been reported to the best of our knowledge.

In our previous work, we discovered that ion–dipole interactions between strong molecular dipole and cation is an effective driving force to stabilize ionic species^30,31^. For example, our group created a solid, aquatically stable ionic conductor without any encapsulations employing the ion–dipole interactions between CF_3_ groups and imidazolium cations. Moreover, the dynamic nature of ion–dipole interactions provides the composite self-healing capability. However, due to the plasticizing effect of ionic liquid, the mechanical property of such a material is weak and the Young’s modulus is only several hundred kilopascal, which cannot meet the demands for practical applications.

Due to the positively charged nature of perovskite QDs, we expect the ion–dipole design can offer an effective and excellent compatibility towards highly stable, stretchable and self-healable polymer composite. Herein, we reported a luminescent perovskite-polymer matrix with good optical transparency, high mechanical strength, excellent stability against all types of harsh environments and self-healable capability in aquatic conditions. Such material has fracture stress of 1.8 MPa and can be stretched to 1300% of its original length, comparable with conventional strong rubbery materials. Notably, their mechanical and optoelectronic properties can remain stable after being immersed in all types of harsh environments (water, strong acid, strong base, and salty water) for months. In addition, they can also self-heal without any external stimuli when damaged in these environments.

## Results and discussion

### Design principle and structural analysis

**Fig. 1:**
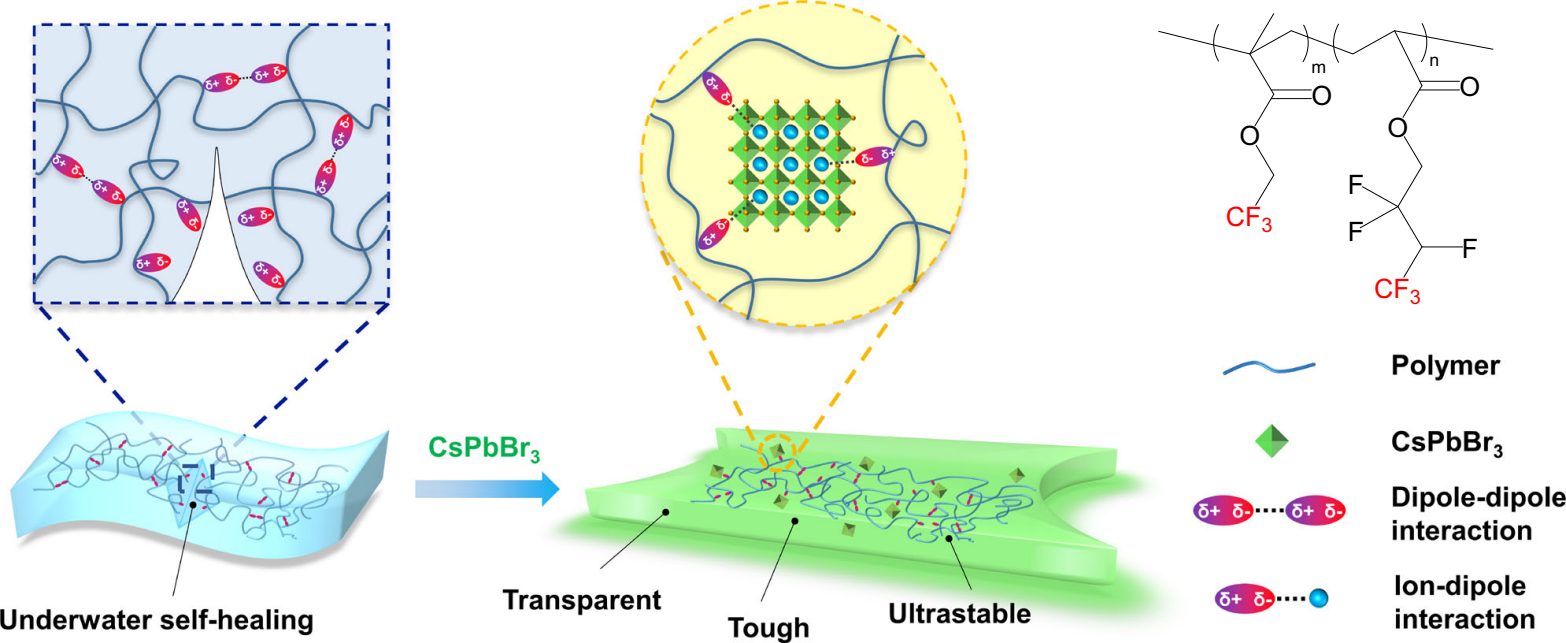
The design of TFE-HF-QD. Schematic illustration of the stretchable, stable and self-healable luminescent perovskite-polymer material.

**Fig. 2:**
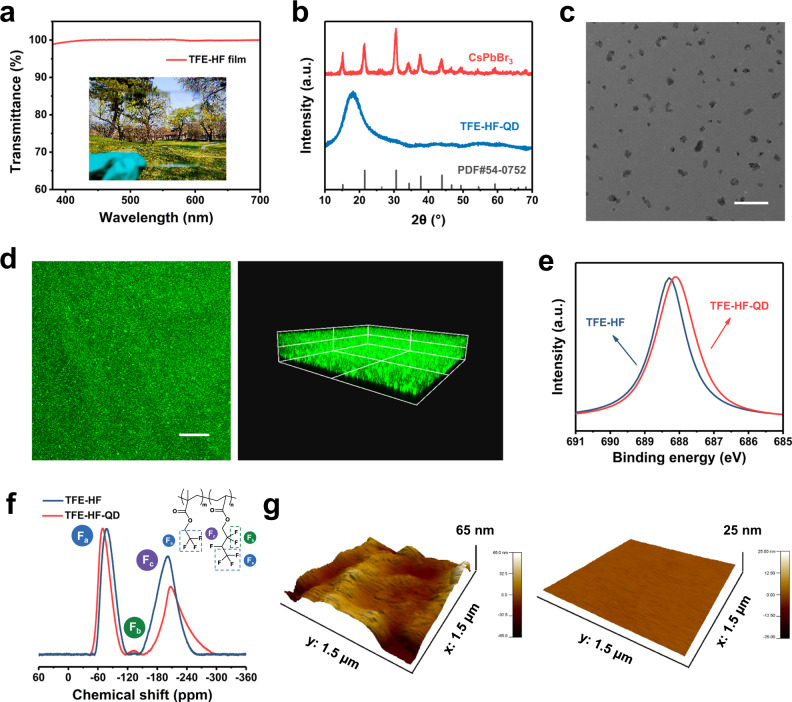
Structural characterization of TFE-HF and TFE-HF-QD. **a** Transmittance spectra of TFE-HF film (0.2 mm) in the photo. **b** The XRD patterns of CsPbBr_3_, TFE-HF-QD and the standard patterns of pure CsPbBr_3_. **c** A typical TEM image of TFE-HF-QD. Scale bar, 0.5 μm. **d** Fluorescence images of TFE-HF-QD film with an excitation wavelength of 488 nm. Scale bar for 2D image, 200 μm. **e** F 1 s spectra of pure TFE-HF and TFE-HF-QD composite films. **f** ^19^F SSNMR spectra of pure TFE-HF and TFE-HF-QD composite films. **g** AFM images of the CsPbBr_3_ film (left) and TFE-HF-QD film (right).

We firstly designed and synthesized an all-dipole fluorine elastomer using the copolymer of 2,2,2-trifluoroethyl methacrylate (TFEMA) and 2,2,3,4,4,4-hexafluorobutyl acrylate (HFBA). Fluorinated polymers usually have high transparency due to their low refractive index^32,33^. Additionally, they are hydrophobicity and chemical stability in aqueous conditions because C–F bond is a very poor hydrogen bonding acceptor^34,35^. The highly polar CF_3_ groups may also interact with each other to facilitate the self-healing process^36^. In our polymer system, The TFEMA polymer chain is rigid while the HFBA polymer chain is relatively soft. The copolymers combination of these two moieties lead to a tough elastomer, we named it as TFE-HF. Most importantly, all the monomers, including TFEMA and HFBA, contain CF_3_ dipoles at the end. In this way, we are able to obtain a fluorine elastomer with super-high density of CF_3_ dipoles to maximize the dipole–dipole and ion–dipole interactions. CsPbBr_3_ QDs were synthesized as reported in the literature^37^. We then directly mixed our monomers with the QDs, they could form a uniform dispersion. The mixture was then directly photo-crosslinked into a transparent elastomer (TFE-HF-QD, Fig. 1). As shown in Fig. 2a, TFE-HF_1.0_ film has a high transparency. The average transmittance of a 0.2 mm thick film is >99% at the visible light region (380–700 nm) and there were no absorption peaks (Supplementary Fig. 1). This highly transparency was important for the light generated by the QDs to easily penetrate the polymer matrix. Gel permeation chromatography was employed to measure the molecular weight of TFE-HF. The results were shown in Supplementary Table 1, Supporting Information.

We first studied the structures of the elastic composite. As shown in Fig. 2b and Supplementary Fig. 2, X-ray diffraction (XRD) and transmission electron microscopy (TEM) results confirmed that the CsPbBr_3_ exhibited a cubic structure with a size of ≈10 nm. After the formation of the TFE-HF-QD composite, the peaks of CsPbBr_3_ disappeared and only left a broad peak at 18°, which ascribed to TFE-HF polymer (Supplementary Fig. 3). The characteristic peaks for CsPbBr_3_ were disappeared, indicating CsPbBr_3_ were encapsulated well by polymer matrix. TEM results of the composite showed that CsPbBr_3_ (dark spots) were uniformly dispersed in the polymer matrix (Fig. 2c). In order to further prove the uniform dispersion of CsPbBr_3_, confocal microscope was also employed. The images were captured using a 10× objective. The two-dimensional (2D) fluorescence image of the film (Fig. 2d, left) proved that CsPbBr_3_ were uniformly distributed in the polymer matrix. The three-dimensional (3D) fluorescence image (Fig. 2d, right) showed high uniformity as well, which agreed well with the TEM results.

The excellent compatibility derived from the strong ion–dipole interactions between CF_3_ dipoles on the polymers and the positively charged QDs could be monitored using X-ray photoelectron spectroscopy (XPS). As shown in Fig. 2e, the F 1 s peak displayed a 0.3 eV shift at the binding energy from 688.4 eV to 688.1 eV after complexation, implying the existing interaction between the electronegative CF_3_ groups and the Cs^+^ and Pb^2+^ cations in CsPbBr_3_^38,39^. Solid-state nuclear magnetic resonance (SSNMR) was further employed to examine ion–dipole interactions. After the introduction of QDs, the singlet at −77.1 ppm in ^19^F SSNMR spectra which attributed to terminal CF_3_ groups in TFE-HF shifted to −69.5 ppm in TFE-HF-QD (Fig. 2f). This was because the interactions between cations and CF_3_ groups reduced the electron density on F atoms so the shielding effect was weakened. The ion–dipole interactions made the CsPbBr_3_ distribute uniformly in the polymer and prevented them from aggregating. For comparison, in the copolymer without fluorine which is composed of ethyl methacrylate (EMA) and butyl acrylate (nBA), it showed no peak shifting in XPS C 1 s and O 1 s spectra (Supplementary Fig. 4). This indicated no ion–dipole interactions existed between the material and QDs. The absence of this interaction made the size of QDs in EMA-nBA-QD was larger than that in TFE-HF-QD (Supplementary Figs. 5, 6). We conducted AFM measurement to explore the topographical features of pristine QDs and encapsulated QDs. As shown in Fig. 2g, TFE-HF-QD films had a smoother surface than pristine CsPbBr_3_ films: the root-mean-square roughness (R_q_) for CsPbBr_3_ and TFE-HF-QD films was 13.6 nm and 0.26 nm, respectively, measured on a surface area of 2.25 μm^2^. The topographical features could also be verified by the SEM image of pure QDs and TFE-HF-QD film (Supplementary Fig. 7). We expect that the uniform matrix can possess excellent mechanical properties as well as high stability.

### Characterization of mechanical properties

**Fig. 3:**
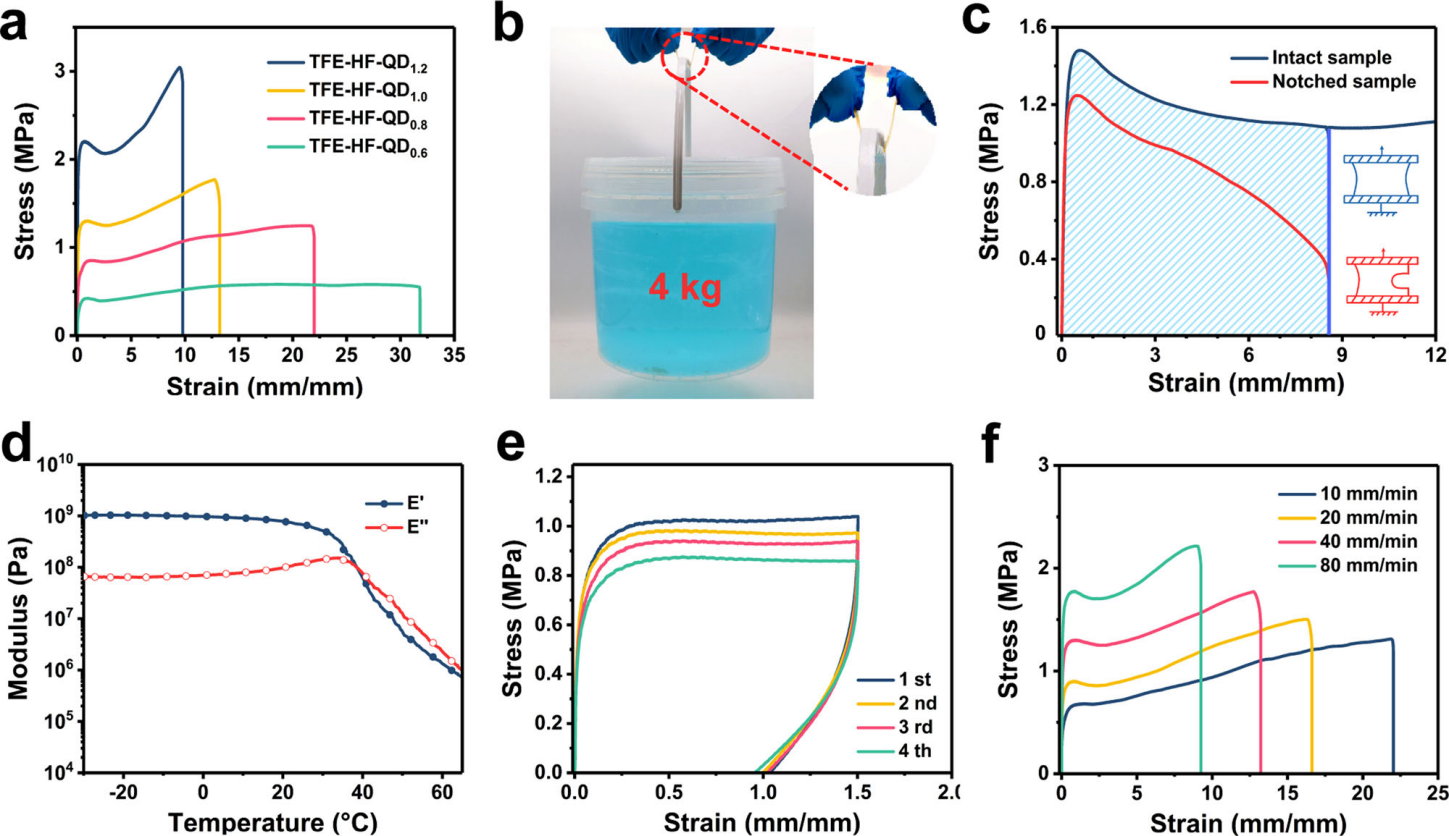
Mechanical properties of TFE-HF-QD. **a** Stress–strain curves of various TFE-HF-QD_*x*_ elastomers. **b** A demonstration of the mechanical strength of TFE-HF-QD_1.0_. **c** The fracture energy of TFE-HF-QD_1.0_. **d** The storage modulus (E') and loss modulus (E'') of TFE-HF-QD_1.0_ elastomer as a function of temperature. **e** Cyclic loading and recovery of TFE-HF-QD_1.0_. **f** Stress-strain curves for TFE-HF-QD_1.0_ samples when stretched at different rates.

The mechanical properties of the TFE-HF-QD matrix were studied using tensile tests. A series of the materials were synthesised and denoted as TFE-HF-QD_*x*_ where *x* is the molar ratio of TFEMA to HFBA, the amount of QDs were kept at 1.0%. As shown in Fig. 3a, TFE-HF-QD_1.0_ could be stretched to 1300% with a fracture stress of 1.8 MPa. As increasing the content of TFEMA, TFE-HF-QD_1.2_ showed a higher fracture stress of 3.0 MPa but lower stretchability (950%). For TFE-HF-QD_0.8_ and TFE-HF-QD_0.6_, they possessed higher strain (excess 2200 and 3200%, respectively) but lower fracture stress (1.2 MPa and 0.5 MPa, respectively). We chose TFE-HF-QD_1.0_ as models in our study because of its balanced mechanical properties for practical applications. Calculated from the tensile-strain curves, the TFE-HF-QD_1.0_ matrix possessed a Young’s modulus of 29 MPa, around 14 times higher than natural rubbers. Therefore, we could use a small TFE-HF-QD_1.0_ sample (2 mm thick and 10 mm in width) to lift a 4 kg of water bucket without breaking (Contact area: 10 × 5 mm, Fig. 3b). The TFE-HF-QD_1.0_ was mechanically tough at the same time. Its fracture energy could reach as high as 30 kJ m^−2^, around 5 times higher than natural rubbers^40^ (Fig. 3c).

The TFE-HF-QD_1.0_ material was also elastic. The temperature-dependent oscillation rheology curve in Fig. 3d showed that the storage modulus (E') of the material was always higher than the loss modulus (E'') at room temperature, indicating the elastic nature. As the molar ratio of soft segments HFBA increased, storage modulus at room temperature showed decreasing tendency. The peak of tan*δ* also moved to lower degrees, indicating a lower *T*_*g*_ value (Supplementary Fig. 8). Cyclic tensile tests were further performed to confirm the material’s robustness and elasticity. The sample was stretched to 1.5 times of its original length and then released for four cycles (Fig. 3e). Each cycle was performed after 30 min of relaxation. The Young’s modulus also remained unchanged after these cycles. If the sample was applied with a larger strain (~1000%), it could also be recovered to its original length after a long time (24 h) of relaxation (Supplementary Fig. 9). The cycling stretching curves almost overlapped for several stretching cycles (strain rate: 10 mm/min, Supplementary Fig. 10). It should be noted that the material also behaved typical rate-dependent mechanical properties (Fig. 3f). At higher loading rates, the composites showed higher stiffness and smaller strains at break. Creep experiments were carried out to investigate the elasticity of TFE-HF-QD_1.0_. As shown in Supplementary Fig. 11, a steady creep rate was reached under a constant stress of 0.3 MPa. When the stress was released, the strain of TFE-HF-QD_1.0_ recovered 75% and a minor permanent deformation remained. All these observations are typical for supramolecular rubbery materials.

### Optical property of TFE-HF-QD_1.0_ under different conditions

**Fig. 4:**
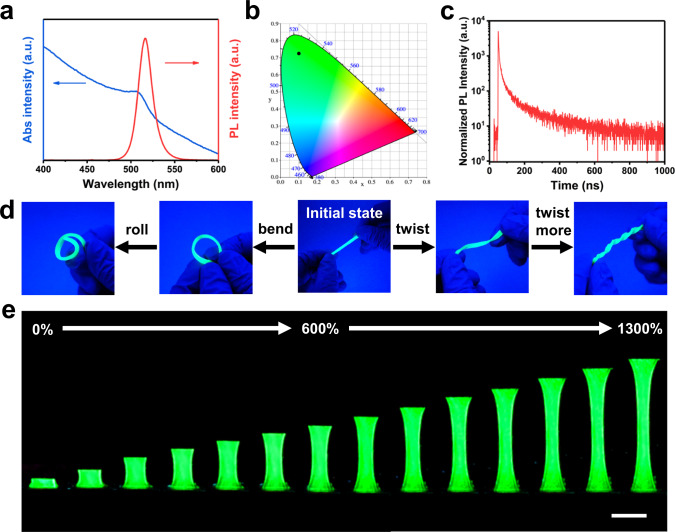
Optical properties and light-emitting performance of TFE-HF-QD_1.0_. **a** The UV–vis absorption (blue) and PL emission (red) spectra of TFE-HF-QD_1.0_. **b** Commission Internationale de L’Eclairage (CIE) coordinates for TFE-HF-QD_1.0_. **c** PL decay dynamics of TFE-HF-QD_1.0_. **d** Photographs of the light-emitting performance of TFE-HF-QD_1.0_ under various deformations: bending, rolling and twisting. **e** Photographs of the light-emitting performance under strain. Scale bar, 1 cm.

Apart from its strong and tough mechanical properties, the TFE-HF-QD_1.0_ also demonstrated excellent luminescence. Figure 4a showed typical photoluminescence (PL) emission and UV–vis absorption spectra. The PL emission wavelength at 521 nm matched very well with the absorption wavelength located in the same range. The FWHM of TFE-HF-QD_1.0_ was as narrow as 19 nm, indicating the high purity of the generated green colour. The pure green emission was also confirmed by Commission Internationale de L’Eclairage (CIE) diagram (Fig. 4b) as the colour coordinated of obtained TFE-HF-QD_1.0_ was (0.1009, 0.7266). The PL lifetimes of TFE-HF-QD_1.0_ was measured by time correlated single photon counting analysis. The PL decay curve over time could be described well by a tri-exponential model:

1 $${{{{{\rm{I}}}}}}\left({{{{{\rm{t}}}}}}\right)={{{\gamma }}}_{0}+{{{{{{\rm{A}}}}}}}_{1}\,{{\exp }}\left(-\frac{{{{{{\rm{t}}}}}}}{{\tau }_{1}}\right)+{{{{{{\rm{A}}}}}}}_{2}\,{{\exp }}\left(-\frac{t}{{\tau }_{2}}\right)+{{{{{{\rm{A}}}}}}}_{3}\,{{\exp }}(-t/{\tau }_{3})$$

which revealed a fast-decay component (time, τ_1_; fraction, f_1_), an intermediate-decay component (time, τ_2_; fraction, f_2_) and a slow-decay component (time, τ_3_; fraction, f_3_). The average PL lifetimes (τ_ave_) of the sample were calculated according to:

2 $${{{\tau }}}_{{{{{{\rm{ave}}}}}}}=\Sigma {{{{{{\rm{A}}}}}}}_{{{{{{\rm{i}}}}}}}{{{\tau }}}_{{{{{{\rm{i}}}}}}}^{2}/\Sigma {{{{{{\rm{A}}}}}}}_{{{{{{\rm{i}}}}}}}{\tau }_{{{{{{\rm{i}}}}}}}$$

According to the data extracted from the fitted curves (Fig. 4c and Supplementary Table 3), τ_1_ of 1.67 ns accounting for 44.49%, τ_2_ of 9.62 ns accounting for 36.88%, and τ_3_ of 88.02 ns accounting for 18.63%. The average PL lifetime was 20.59 ns.

Next, the optical property of TFE-HF-QD_1.0_ under different mechanical deformations was investigated. It maintained a bright green emission after bending and twisting (Fig. 4d). Further bending and twisting still could not affect its emission performance, indicating the optical property was robust against various mechanical deformations. The stability of light emission was also confirmed by stretching TFE-HF-QD_1.0_ with large strain. As can be seen in Fig. 4e and Supplementary Movie 1, it exhibited a uniform, bright emission over the entire sample in the original state and retained until an extremely large strain of 1300%. Thus, TFE-HF-QD_1.0_ will show excellent sustainable light-emitting performance in various extreme mechanical deformations owing to their mechanical robustness.

### A stable material against all types of harsh environments

**Fig. 5:**
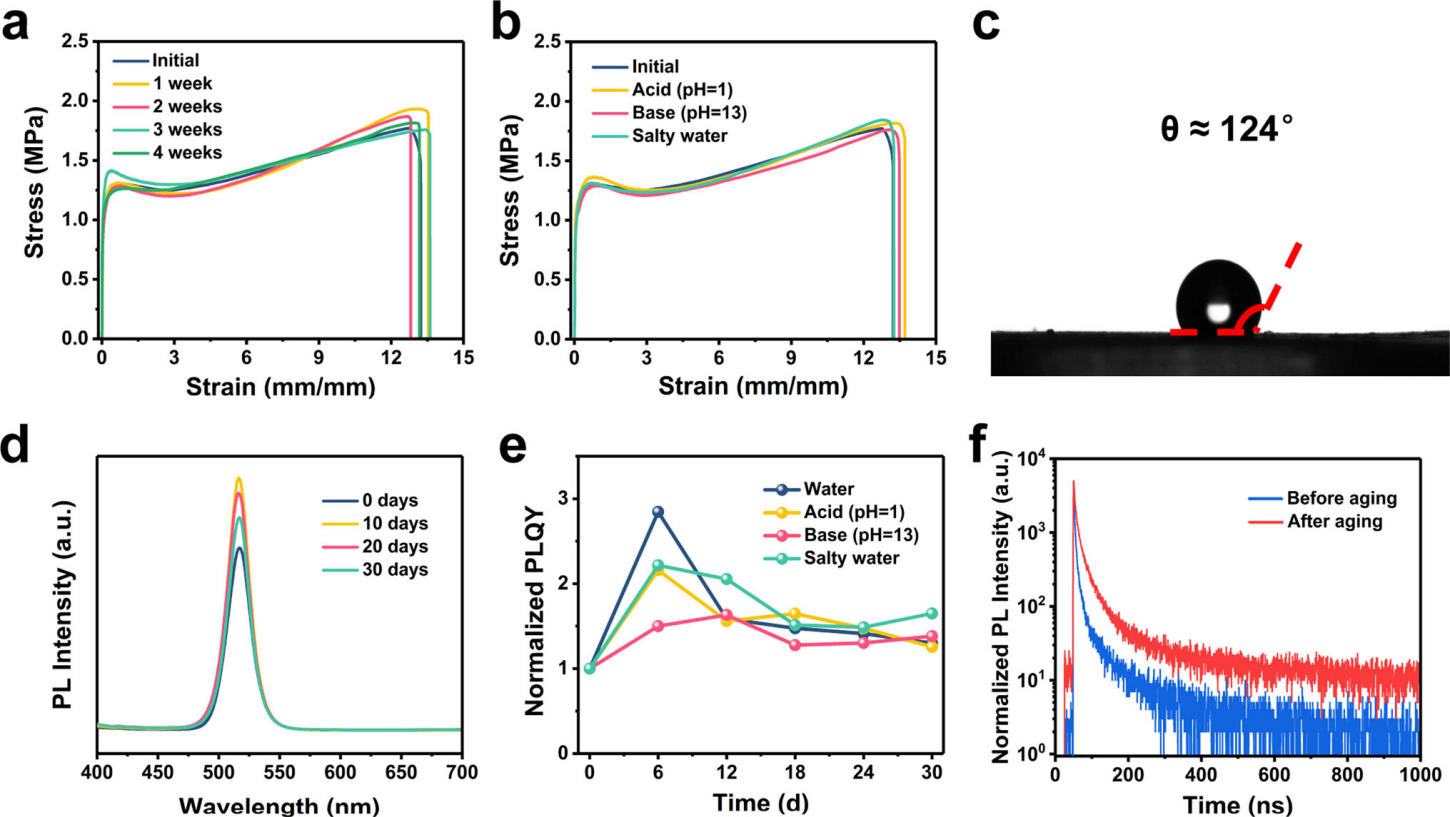
Stability of TFE-HF-QD_1.0_ under different conditions. Mechanical properties of TFE-HF-QD_1.0_ after immerged in **a** water for 4 weeks and **b** different harsh aqueous environments for a month. **c** Contact angle of water of TFE-HF-QD_1.0_. **d** PL intensity of TFE-HF-QD_1.0_ after being immersed in water for different times. **e** Variation of normalized PLQY as a function of time. **f** PL decay dynamics of TFE-HF-QD_1.0_ before and after ageing in water.

Notably, the mechanical properties and photo-luminescent properties of TFE-HF-QD_1.0_ are highly stable against all types of harsh environments. We chose pure water, hydrochloric acid (HCl) solutions (pH = 1), sodium hydroxide (NaOH) solutions (pH = 13) and salty seawater as model conditions for our study. The TFE-HF-QD_1.0_ samples were immersed in water for 1 month. Their mechanical and photo-luminescent properties were recorded each week. As shown in Fig. 5a, the stress–strain curves almost overlapped with each other after 4 weeks’ storage under water. It’s more impressive to see that the mechanical property of our material was highly stable in other harsh environments as well after 1 month’s storage (Fig. 5b). This hydrophobic characteristic originated from the fluorinated polymer backbone as convinced by the contact angle of water (Fig. 5c). It could reach 124° compared to that of PDMS. Then we evaluated the optical stability of TFE-HF-QD_1.0_. A sample with THU letters was prepared (Supplementary Fig. 12). It could still emit a bright green light under UV excitation through the entire body after being immersed in water for a month. We tracked their PL emission intensity as a function of time and found it maintained stable after immersed in water (Fig. 5d), indicating encapsulating the QDs in hydrophobic polymer matrix could effectively improve their stability against high humidity or even aqueous solutions. The PL intensity of the samples which immersed in HCl solution, NaOH solution and salty water were also recorded (Supplementary Fig. 13). All of them exhibited stable photoluminescence after 1 month. The photo-luminescent quantum yields (PLQY) were recorded as well (Supplementary Table 2). As revealed by Fig. 5e, the normalized PLQY value (PLQY/PLQY_initial_) kept stable without obvious decay after 1 month of immerging. The increase of PLQY at the beginning was probably attributed to surface passivation of a minor amount of water^41,42^. This was also verified by the comparison of PL lifetimes before and after aging in water and other aqueous conditions (Fig. 5f, Supplementary Fig. 14 and Supplementary Table 3). The average PL lifetime after ageing in water was 43.19 ns, around twice of that before ageing, indicating a decrease in the number of surface defects. The superior stability might come from abundant −CF, −CF_2_, and −CF_3_ groups on the polymer backbones. The high electronegativity of fluorine and the strong electrostatic nature of the C–F bond render organic fluorine a poor donor and hydrogen bonding acceptor^43–45^. Therefore, C–F bond will not interact with water molecules and the material can keep stable in water without mechanical and optical decay for a long time. Notably, our material also possessed stability against organic solvent such as hexane. Compared to conventional stretchable luminescent elastomer, for example, SEBS, TFE-HF-QD_1.0_ could still be free-standing and emitted green light after being immersed in hexane for 2 weeks (Supplementary Fig. 15). On the contrary, SEBS became a sticky, gel like material and could not free stand (Supplementary Fig. 16).

### Underwater self-healing property and mechanism

**Fig. 6:**
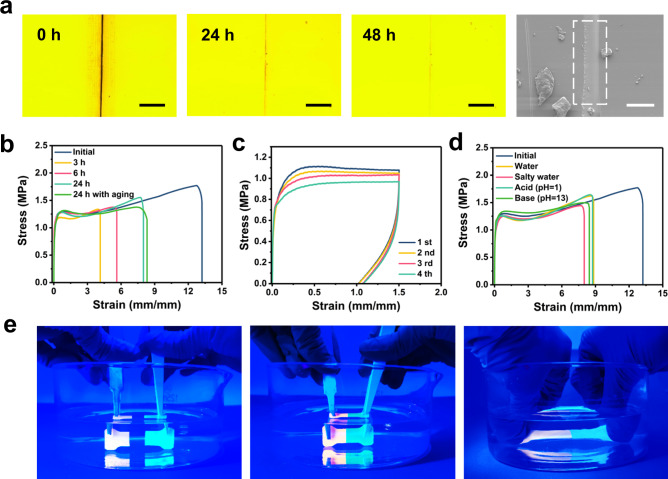
Self-healing performance of TFE-HF-QD_1.0_. **a** Optical and SEM image of TFE-HF-QD_1.0_ before and after healing. Scale bar of optical images, 200 μm. Scale bar of SEM image, 50 μm. **b** Stress–strain curve of TFE-HF-QD_1.0_ after healing in air. **c** Stress–strain cycling curves of the cut TFE-HF-QD_1.0_ samples with the interval 30 min. **d** Stress–strain curve of TFE-HF-QD_1.0_ after healing under different aqueous conditions. **e** Cut samples healed underwater and stretched after healing.

The capability to heal the damage and restore the mechanical strength was important for TFE-HF-QD_1.0_ to serve as underwater wearable/stretchable devices and cloths. It possessed a large amount of ion–dipole and dipole–dipole interactions and the movement of polymer chains was less restrained due to low *T*_*g*_ (Supplementary Fig. 17), which contributed to the self-healing process. The healing process could be observed by a microscope. The samples were cut in half with a fresh razor blade and put together closely at ambient temperature. As shown in Fig. 6a, the scratch became lighter after 24 h and almost disappeared after 48 h. The good healing site could also be viewed by SEM. Mechanical test was employed to measure the healing efficiency (*η*) of TFE-HF-QD_1.0_, which *η* is defined as the proportion of restored toughness relative to the original one. Figure 6b showed the healing efficiencies of the materials in ambient air. After healing for 6 h, the material could be stretched to >500% with a healing efficiency of 37.65%. After healing for 24 h, the healing efficiency could reach 55.00%. The elasticity of healing samples also recovered just like the initial ones (Fig. 6c). Interestingly, we found the healing had no obvious ageing effect. As the cut sample was stored in air for 6 h and put together, they could still self-heal and the healing efficiency could reach 55.78% compared to that without aging. Prolonged the ageing time to 12 h and 24 h, TFE-HF-QD_1.0_ could still self-heal (Supplementary Fig. 18). Since the water had very limited effect on the bonding constant of dipole–dipole interactions^46^, TFE-HF-QD_1.0_ could also self-heal under different aqueous environments, including water, salty water, acidic solution (pH = 1), and basic solution (pH = 13). All self-healing efficiency values were compared to that in the air (Fig. 6d). A sample stained with Rhodamine B and TFE-HF-QD_1.0_ were cut into halves and put together in water. The healed sample could be stretched several times of its original length after healing for 24 h (Fig. 6e). This self-healing capability guaranteed the material’s service life and made it a reliable underwater wearable/stretchable material.

**Fig. 7:**
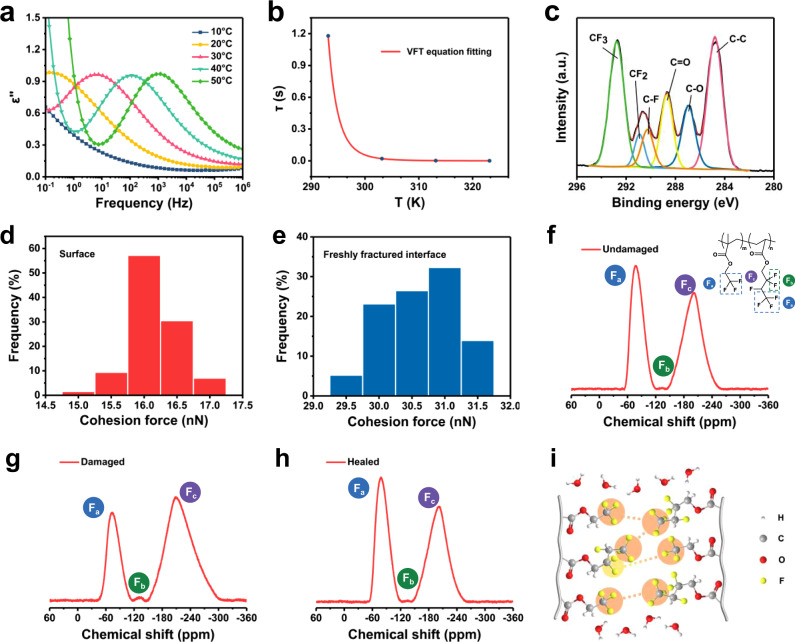
Self-healing mechanism. **a** Imaginary permittivity (ε'') of TFE-HF-QD_1.0_ as a function of frequency from 10 °C to 50 °C. **b** Relaxation time (τ) of TFE-HF-QD_1.0_ obtained from the fitted VFT equation. **c** XPS spectra of C 1 s region of freshly fractured interface. Cohesion force of **d** surface and **e** freshly fractured interface of TFE-HF-QD_1.0_. ^19^F SSNMR spectra of **f** undamaged, **g** damaged and **h** healed sample. **i** Schematic illustration of the self-healing process of TFE-HF-QD_1.0_.

As known that the self-healing ability was related to the motion of the polymer segments. When two separated parts came together, the diffusion and entanglement of polymer chains helped to heal the material^47,48^. For the self-healing mechanism, we first conducted a broadband dielectric spectrum to study the molecular dynamics of the material. The test frequency swept from 10^−1^ Hz to 10^6^ Hz at temperatures from 10 °C to 50 °C. As shown in Fig. 7a, an obvious peak attributed to α relaxation was observed in the imaginary permittivity (ε'') spectrum, which represented the segmental motion of the polymer chains. The relaxation time (τ) was calculated according to the following equation:

3 $${{\tau }}=1/2{\pi {{{{{\rm{f}}}}}}}_{{{{{{\rm{p}}}}}}}$$

where f_p_ was the frequency corresponding to the peaks of ε''. The τ values were fitted based on the Vogel-Fulcher-Tammann (VFT) equation (Fig. 7b). The results showed the relaxation time ranged from 1 s to 10^−4^ s at the temperature from 20 °C to 50 °C, indicating a fast diffusion dynamic of polymer segments. Thus ensured the self-healing property of the polymer. At high temperatures, the polymer chains moved intensely so the τ values became shorter, which made the self-healing process faster. This was also convinced by the stress–strain curve of the sample healing at 40 °C (Supplementary Fig. 19). The healing efficiency could reach nearly 100% after 24 h.

In order to deeply understand the underlying self-healing mechanism, XPS was employed to investigate the molecular configuration of the freshly fractured interface. As depicted in Fig. 7c, the C 1 s region for the interface was fitted to six peaks, which assigned to CF_3_ (292.7 eV), CF_2_ (290.9 eV), C–F (290.2 eV), C = O (288.6 eV), C–O (286.9 eV) and C–C (284.8 eV), respectively. In molecular solids containing both H and F atoms, the rotational energy of fluoromethyl (CF_3_) groups is higher than methyl (CH_3_) groups so CF_3_ groups contributed more to the chain conformational change during the self-healing process^49^. We thought the abundant dipole–dipole interactions at the interface together with the easy segmental mobility increased the probability of polymer mutual entanglement at the fracture place, thus promoted the healing process of the material. To further confirm the role CF_3_ groups played in the self-healing process, we used atomic force microscope to measure the cohesion force for the surface and freshly fractured interface of TFE-HF-QD material. The results showed that the freshly fractured surface had a cohesion force of 29.5–31.5 nN derived from numerous dipole–dipole interactions (Fig. 7e), approximately twice higher than that of surface (Fig. 7d)^50,51^. The increment of cohesion force indicated these strong polar groups could form dipole–dipole interactions and facilitate the self-healing process.

We also employed solid state nuclear magnetic resonance (SSNMR) to examine interactions between CF_3_ groups in different samples. Figure 7f–h showed ^19^F SSNMR spectra of undamaged, damaged and self-healed TFE-HF-QD samples, respectively. A singlet at −77.1 ppm was attributed to terminal CF_3_ groups. Here we named it peak *F*_a_. The peaks at −131.9 ppm and −201.3 ppm were attributed to CF_2_ and CF groups, respectively, which was peak F_b_ and peak F_c_. In the undamaged sample, the ratio of peak *F*_a_ area integral to peak *F*_b_ and *F*_c_ area integral was 0.83. Upon mechanical damage, the ratio changed to 0.40. As the ^19^F was considerably more sensitive to local magnetic environment than ^1^H, the decrement of the area integral ratio in 1D ^19^F NMR upon damage was attributed to the shortening of T_2_ relaxation times, indicated after mechanical damage, the mobility of the CF_3_ groups was restricted^52,53^. After healing, the ratio increased to 0.85, compared to that of the undamaged sample.

Based on these characterizations, we supposed the self-healing process of TFE-HF-QD was as follows: When the material was damaged, the system changed from a relatively stable state to an energetically unfavourable state. The strong interaction between highly polar CF_3_ groups at the interface altered the polymer chain conformations to reduce the entropy effect. The long chains were compressed and shortened the interchain distance thus causing the decrease of T_2_ relaxation times. As the segments healed and recovered to their stable state, the chains were decompressed and increased interchain distances so leading to the increase of T_2_ relaxation times. In addition, the abundant F element formed hydrophobic surfaces favoured the formation of a water-resistant molecular bridge between the fractured parts^54^, which endowed the material with outstanding underwater self-healing behaviour (Fig. 7i).

### Potential applications

**Fig. 8:**
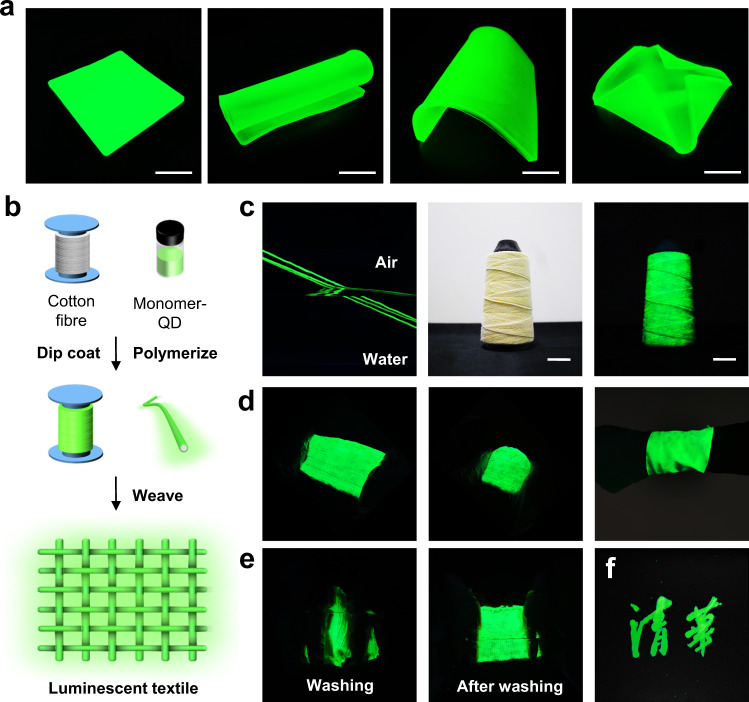
Potential applications of TFE-HF-QD_1.0_. **a** A large dimensional TFE-HF-QD_1.0_ film was prepared. It could emit light under different deformations. Scale bar, 5 cm. **b** Schematic illustration for the preparation of underwater luminescent textile. **c** Luminescent fibre could keep stable both in air and water (left). Photographs of TFE-HF-QD_1.0_ cotton fibre on a spool (middle and right). Scale bar, 2.5 cm. **d** A large dimension luminescent cotton fabric (left and middle). It could be used as a wristband (right). **e** Washing process of the luminescent textile. **f** A hand-writing luminescent logo.

As mentioned above, such a facile photo-polymerization technique provided an opportunity to fabricate luminescent devices in large dimensions. As shown in Fig. 8a, a green emissive TFE-HF-QD_1.0_ plate with a dimension of 10 × 10 × 1 cm was successfully and conveniently prepared. It maintained a bright green emission after bending and folding. Apart from easy fabrication, the combination between high mechanical strength, excellent stability and self-healable capability made TFE-HF-QD_1.0_ a superior candidate for multiple water-prone applications such as aquatic wearable/stretchable displays and water-resistant devices. A luminescent cotton fibre was prepared as shown in Fig. 8b. The luminescent fibres could be easily fabricated by dip-coating the cotton fibres in the QDs and monomer mixture solution for a few seconds and then polymerized. During the soaking process, the cotton fibres could take in sufficient QDs and monomer. This solution-based coating is a simple way to obtain continuous lengths of luminescent fibres (Fig. 8c). It can emit a bright green light both in air and water due to the high stability of TFE-HF-QD_1.0_.

Next, using this luminescent fibre we could conveniently weave a large luminescent textile. As shown in Fig. 8d, a green emissive TFE-HF-QD_1.0_ textile with a dimension of 20 × 10 cm was prepared. Bending, folding and stretching the textile would not affect its optical property (Supplementary Movie 2) so it could be used as a luminescent wrist band to act as an underwater communication medium. In addition, due to its high stability towards water, it could even keep stable after hand-washing process (Fig. 8e and Supplementary Movie 3). This implies a potential application of TFE-HF-QD_1.0_ textile in aquatic wearable water-resistant luminescent clothes. Apart from the dip-coating process, a luminescent logo was also prepared by hand-writing method. We dipped a Chinese brush in the QDs-monomer mixture solution and wrote on the textile. Then the pattern was polymerized for some time. As shown in Fig. 8f, the Chinese character of Tsinghua University was presented on the fabric and could keep stable underwater to work as a luminescent logo. We hope this new kind of stable photo-luminescent material has huge potential in aquatic wearable/stretchable displays and water-resistant devices.

## Conclusion

In summary, we prepare an all-dipole fluorine luminescent elastomer with good optical transparency, high mechanical strength, excellent stability against all harsh aqueous environments and self-healable capability in aquatic conditions. Due to the strong ion–dipole interactions between CF_3_ dipoles on the polymers and the positively charged QDs, such a material possessed an excellent compatibility. Their mechanical and optical properties can remain stable after being immersed in all types of harsh environments (water, strong acid, strong base and salty water) for months. In addition, they can also self-heal without any external stimuli when damaged in these environments. We believe this new kind of stable photo-luminescent stretchable material has huge potential in optoelectronic devices in marine applications.

## Methods

### Reagents

Lead bromide (PbBr_2_, Xi’an Polymer Light Technology Corp., >99.99%), Caesium carbonate (Cs_2_CO_3_, Energy Chemical, 99.9%), Oleic acid (OA, Energy Chemical, 90%), Oleylamine (OAm, Energy Chemical, 80−90%), 1-Octadecene (ODE, Energy Chemical, 90%), Toluene (Beijing Tongguang Fine Chemical Company, AR), Hexane (Greagent, AR, ≥97.0%), 2,2,2-Trifluoroethyl methacrylate (TFEMA, Aladdin, 98%), 2,2,3,4,4,4-Hexafluorobutyl acrylate (HFBA, Aladdin, >95%), Ethyl methacrylate (EMA, Aladdin, 99%), Butyl acrylate (nBA, Amethyst Chemicals, 99.5%), 2-Hydroxy-2-methylpropiophenone (HMPP, Energy Chemical, 98%).

### Preparation of Cs-oleate

0.814 g of Cs_2_CO_3_, 2.5 mL OA and 40 mL ODE were loaded into a 100 mL 3-neck flask. The mixture was dried under vacuum for 1 h at 120 °C, and then heated to 150 °C under N_2_ until all Cs_2_CO_3_ reacted with OA. The precursor was stored at 100 °C for further use.

### Synthesis of CsPbBr_3_ perovskite QDs

20 mL ODE, 0.276 g PbBr_2_, 2.5 mL OA, and 2.5 mL OAm were mixed in a 3-neck flask and dried under vacuum at 120 °C for 1 h. Then the temperature was raised to 170 °C under N_2_ environment. After maintaining for 10 min, 1.6 mL of Cs-oleate precursor was quickly injected into the solution, and 5 s later, the flask was placed in an ice-bath and cooled to room temperature. The solution’s colour turned to bright green. The crude solution was centrifuged at 10000 rpm for 10 min and the precipitate was washed with toluene. Then it was centrifuged at 10000 rpm for 10 min. Finally, the precipitate was dispersed in 2 mL hexane and kept at 4 °C.

### Fabrication of stretchable luminescent material

10 mmol TFEMA, 10 mmol HFBA, and 21 mmol HMPP were mixed in a glass bottle A and stirred for 4 h. 10 μL perovskite QDs hexane solution was put in another glass bottle B and evaporated the hexane at room temperature. Then the monomer solution was poured into glass bottle B. The mixture was sonicated for 1 min to make the QDs disperse uniformly, then was injected into a Teflon mould for UV photo-polymerization for 1 h. Finally, a stretchable luminescent material was obtained. For TFE-HF-QD_1.2_, TFE-HF-QD_0.8_ and TFE-HF-QD_0.6_ samples, the molar number of TFEMA was changed to 12 mmol, 8 mmol, and 6 mmol, respectively. The TFE-HF samples were polymerized without QDs.

### Synthesis of EMA-nBA-QD

10 mmol EMA, 10 mmol nBA, and 21 mmol HMPP were mixed in a glass bottle A and stirred for 4 h. 10 μL perovskite QDs hexane solution was put in another glass bottle B and evaporated the hexane at room temperature. Then the monomer solution was poured into glass bottle B. The mixture was sonicated for 1 min to make the QDs disperse uniformly, then was injected into a Teflon mold for UV photo-polymerization for 1 h. EMA-nBA was synthesized in the same way without QDs.

### Characterizations

Transmission data was obtained using HITACHI UH4150 spectrophotometer. Measurements were taken between 380 and 700 nm with a resolution of 1 nm. The contact angle data of water droplets were collected by a drop shape analyzer (Dataphysics OCA15Pro) at ambient temperature. The volume of water droplet was 2 μL. Films for characterization of transmittance and water contact angle were prepared by polymerizing the monomer on a glass slide (50 mm × 50 mm). ^19^F NMR spectroscopic measurements were conducted on 600 MHz JEOL-ECZ600R spectrometer with 5 s relaxation delay and each spectrum represents 8 added scans. NMR samples were prepared by UV polymerization in a PTFE mould. Healed samples were allowed to heal at 25 °C for 72 h before NMR test. ^19^F NMR for TFE-HF (600 MHz) δ: −77.1 ppm (−CF_3_), −132.5 ppm (−CF_2_), −201.3 ppm (−CF). ^19^F NMR for TFE-HF-QD (600 MHz) *δ*: −69.5 ppm (−CF_3_), −131.9 ppm (−CF_2_), −201.3 ppm (−CF). The confocal PL images were taken from an Olympus FV3000 Confocal Systems using a 10× objective with an excitation wavelength of 488 nm. X-ray diffraction data (XRD) was conducted by Rigaku SmartLab. The diffraction angular 2θ is ranged from 10° to 70° at an increment of 2° min^−1^. The diffractometer system uses Cu tube as an X-ray source with an intensity of 150 mA and a tension of 40 kV. X-ray photoelectron spectroscopy (XPS) were recorded on a PHI Quantro SXM X-ray photoelectron spectrometer with monochromatized Al *K*α X-ray source (hν = 1253.6 eV, 10 mA, 15 kV). The spectra were recorded by 0.05 eV step. All the sample preparation, storage, and transfer operations were isolated from moisture and oxygen.

To observe the surface topology of the samples, SEM method was performed. Before running the system, all samples were vacuumed up to ~1 Pa and were sputtered with a thin gold-palladium layer in 210 s (Quorum-Q150R). The surface morphologies were monitored using a HITACHI SU-8010 SEM device. AFM images were recorded on an Oxford Cypher VRS with OMCL-AC240TS Si tips, cohesion force was measured with Si tip coated with TFE-HF polymers. The morphology of perovskite quantum dots and its dispersity in polymers were inspected using transmission electron microscopy (HT7700, HITACHI) operated at 200 kV. Before performing TEM test, the samples were prepared by spin-coating the QDs-monomer solution on the Cu mesh with a spin rate of 1500 r/min for 30 s. Then the Cu mesh was put under UV irradiation to polymerize for 1 h.

Mechanical tests were conducted on Sunstest UTM2502 at room temperature. All the experiments were performed at a strain rate of 40 mm min^−1^ unless otherwise noted. The cyclic tensile stress–strain curves were obtained at the strain rate of 40 mm min^−1^ with the interval of 30 min between adjacent cycles unless otherwise noted. Samples for aqueous self-healing test were additionally dried in vacuum for 4 h at ambient temperature. Young’s modulus was calculated from the initial slope of the stress–strain curves. Each mechanical test was repeated with at least three individual samples.

DMA tests were carried on with TA Instruments DMA 850 at a heating speed of 5 °C min^−1^ from −30 °C to 70 °C. DSC measurements were recorded on a TA Instruments DSC 250 with a heating speed of 5 °C min^−1^ from −40 °C to 40 °C. Dielectric properties were measured by a Broadband Dielectric Spectrometer (Alpha-T, Novocontrol Technologies GmbH & Co. KG). The circular sheet with the diameter of 10 mm and thickness of 400 μm was coated with Pt particles and placed into two parallel electrodes. The frequency sweep range is from 10^−1^ to 10^6^ Hz.

UV–Vis absorption spectra were measured on a UV-6100 UV–Vis spectrophotometer (Shanghai Mapada Instruments Co. Ltd., China). PL spectra were taken using an F-380 fluorescence spectrometer (Tianjin Gangdong Sci. & Tech. Development. Co., Ltd., China). Time-resolved PL was collected using fluorescence lifetime measurement system (C11367-11, Hamamatsu Photonics, Japan) with an excitation wavelength of 365 nm. The absolute PLQYs were determined using a fluorescence spectrometer with an integrated sphere (C9920-02, Hamamatsu Photonics, Japan) under excited at a wavelength of 365 nm. Optical micrographs were recorded with a cross-polarized optical microscope (Nikon LV100N POL). All the photos were taken by Canon M50.

## Supplementary information

Supplementary Information

Description of Additional Supplementary Files

Supplementary Movie 1

Supplementary Movie 2

Supplementary Movie 3

## Data Availability

All data needed to evaluate the conclusions in the paper are presented in the paper and/or the Supplementary Information. Additional data related to this paper may be requested from the authors.

## References

[1] Swarnkar A (2015). Colloidal CsPbBr_3_ perovskite nanocrystals: luminescence beyond traditional quantum dots. Angew. Chem. Int. Ed..

[2] Lee J, Sundar VC, Heine JR, Bawendi MG, Jensen KF (2000). Full color emission from II–VI semiconductor quantum dot-polymer composties. Adv. Mater..

[3] Liu XK (2021). Metal halide perovskites for light-emitting diodes. Nat. Mater..

[4] Chen, H., Pina, J. M., Hou, Y. & Sargent, E. H. Synthesis, applications, and prospects of quantum‐dot‐in‐perovskite solids. *Adv. Energy Mater*. 10.1002/aenm.202100774 (2021).

[5] Jagielski J (2017). Aggregation-induced emission in lamellar solids of colloidal perovskite quantum wells. Sci. Adv..

[6] Kovalenko MV, Protesescu L, Bodnarchuk MI (2017). Properties and potential optoelectronic applications of lead halide perovskite nanocrystals. Science.

[7] Zou S (2017). Stabilizing cesium lead halide perovskite lattice through Mn(II) substitution for air-stable light-emitting diodes. J. Am. Chem. Soc..

[8] Ravi VK, Scheidt RA, Nag A, Kuno M, Kamat PV (2018). To exchange or not to exchange. suppressing anion exchange in cesium lead halide perovskites with PbSO_4_-oleate capping. ACS Energy Lett..

[9] Yang S (2016). Functionalization of perovskite thin films with moisture-tolerant molecules. Nat. Energy.

[10] Xuan T (2017). Highly stable CsPbBr_3_ quantum dots coated with alkyl phosphate for white light-emitting diodes. Nanoscale.

[11] Swarnkar A (2016). Quantum dot-induced phase stabilization of α-CsPbI_3_ perovskite for high-efficiency photovoltaics. Science.

[12] Zhu H (2017). Organic cations might not be essential to the remarkable properties of band edge carriers in lead halide perovskites. Adv. Mater..

[13] Pu C, Peng X (2016). To battle surface traps on CdSe/CdS core/shell nanocrystals: shell isolation versus surface treatment. J. Am. Chem. Soc..

[14] Malgras V, Henzie J, Takei T, Yamauchi Y (2018). Stable blue luminescent CsPbBr_3_ perovskite nanocrystals confined in mesoporous thin films. Angew. Chem. Int. Ed..

[15] Sun H (2017). Chemically addressable perovskite nanocrystals for light-emitting applications. Adv. Mater..

[16] Wang YK (2021). All-inorganic quantum-dot LEDs based on a phase-stabilized alpha-CsPbI_3_ perovskite. Angew. Chem. Int. Ed..

[17] Solari SF (2021). Ligand-assisted solid phase synthesis of mixed-halide perovskite nanocrystals for color-pure and efficient electroluminescence. J. Mater. Chem. C. Mater..

[18] Liao H (2018). A general strategy for in situ growth of all-inorganic CsPbX_3_ (X = Br, I, and Cl) perovskite nanocrystals in polymer fibers toward significantly enhanced water/thermal stabilities. Adv. Opt. Mater..

[19] Xuan TT (2019). Super-hydrophobic cesium lead halide perovskite quantum dot-polymer composites with high stability and luminescent efficiency for wide color gamut White light-emitting diodes. Chem. Mater..

[20] Cha W, Kim H-J, Lee S, Kim J (2017). Size-controllable and stable organometallic halide perovskite quantum dots/polymer films. J. Mater. Chem. C..

[21] Park JM (2020). Aromatic nonpolar organogels for efficient and stable perovskite green emitters. Nat. Commun..

[22] Xin Y, Zhao H, Zhang J (2018). Highly stable and luminescent perovskite-polymer composites from a convenient and universal strategy. ACS Appl. Mater. Interfaces.

[23] Wei Y (2017). Enhancing the stability of perovskite quantum dots by encapsulation in crosslinked polystyrene beads via a swelling-shrinking strategy toward superior water resistance. Adv. Funct. Mater..

[24] Wang Y (2016). Ultrastable, highly luminescent organic-inorganic perovskite-polymer composite films. Adv. Mater..

[25] Zhang YC (2019). Homogeneous freestanding luminescent perovskite organogel with superior water stability. Adv. Mater..

[26] Liu K, Jiang Y, Bao Z, Yan X (2019). Skin-inspired electronics enabled by supramolecular polymeric materials. CCS Chem..

[27] Li TQ, Wang YT, Li SH, Liu XK, Sun J (2020). Mechanically robust, elastic, and healable ionogels for highly sensitive ultra-durable ionic skins. Adv. Mater..

[28] Li CH, Zuo JL (2020). Self-healing polymers based on coordination bonds. Adv. Mater..

[29] Liu Y, He K, Chen G, Leow WR, Chen X (2017). Nature-inspired structural materials forflexible electronic devices. Chem. Rev..

[30] Cao Y (2019). Self-healing electronic skins for aquatic environments. Nat. Electron..

[31] Zhang Y (2020). Highly transparent, underwater self-healing, and ionic conductive elastomer based on multivalent ion–dipole interactions. Chem. Mater..

[32] Groh W, Zimmermann A (1991). What is the lowest refractive index of an organic polymer?. Macromolecules.

[33] Gaynor J, Schueneman G, Schuman P, Harmon JP (1993). Effects of fluorinated substituents on the refractive index and optical radiation resistance of methacrylates. J. Appl. Polym. Sci..

[34] Xu, L., et al. A transparent, highly stretchable, solvent-resistant, recyclable multifunctional ionogel with underwater self-healing and adhesion for reliable strain sensors. *Adv. Mater*. **33**, e2105306 (2021). 10.1002/adma.202105306 34647370

[35] Yu, Z. C. & Wu, P. Y. Underwater communication and optical camouflage ionogels. *Adv. Mater*. **33**, 2008479 (2021). 10.1002/adma.202008479 33955597

[36] O’Hagan D (2008). Understanding organofluorine chemistry. An introduction to the C–F bond. Chem. Soc. Rev..

[37] Protesescu L (2015). Nanocrystals of cesium lead halide perovskites (CsPbX_3_, X = Cl, Br, and I): novel optoelectronic materials showing bright emission with wide color gamut. Nano Lett..

[38] Zhou Q (2016). In situ fabrication of halide perovskite nanocrystal-embedded polymer composite films with enhanced photoluminescence for display backlights. Adv. Mater..

[39] Yang L, Fu B, Li X, Chen H, Li L (2021). Poly(vinylidene fluoride)-passivated CsPbBr_3_ perovskite quantum dots with near-unity photoluminescence quantum yield and superior stability. J. Mater. Chem. C..

[40] Dragoni E, Medri G (1988). Fracture toughness evaluation of natural rubber. Theor. Appl. Fract. Mech..

[41] Zhou H (2020). Water passivation of perovskite nanocrystals enables air-stable intrinsically stretchable color-conversion layers for stretchable displays. Adv. Mater..

[42] Jang J (2020). Extremely stable luminescent crosslinked perovskite nanoparticles under harsh environments over 1.5 years. Adv. Mater..

[43] Dalvi VH, Rossky PJ (2010). Molecular origins of fluorocarbon hydrophobicity. Proc. Natl Acad. Sci. USA.

[44] Howard JAK, Hoy VJ, O’Hagan D, Smith GT (1996). How good is fluorine as a hydrogen bond acceptor?. Tetrahedron.

[45] Dunitz JD, Taylor R (1997). Organic fluorine hardly ever accepts hydrogen bonds. Chem. Eur. J..

[46] Cao Y (2018). A highly stretchy, transparent elastomer with the capability to automatically self-heal underwater. Adv. Mater..

[47] Wang S, Urban MW (2020). Self-healing polymers. Nat. Rev. Mater..

[48] Yang Y, Ding X, Urban MW (2015). Chemical and physical aspects of self-healing materials. Prog. Polym. Sci..

[49] Beckmann PA, Rheingold AL (2016). ^1^H and ^19^F spin-lattice relaxation and CH_3_ or CF_3_ reorientation in molecular solids containing both H and F atoms. J. Chem. Phys..

[50] Lee H, Scherer NF, Messersmith PB (2006). Single-molecule mechanics of mussel adhesion. Proc. Natl Acad. Sci. USA.

[51] Zeng H, Hwang DS, Israelachvili JN, Waite JH (2010). Strong reversible Fe^3+^-mediated bridging between dopa-containing protein films in water. Proc. Natl Acad. Sci. USA..

[52] Peng H (2009). Synthesis and evaluation of partly fluorinated block copolymers as MRI imaging agents. Biomacromolecules.

[53] Wang, S. & Urban, M. W. Self-healable fluorinated copolymers governed by dipolar interactions. *Adv. Sci.* **8,** e2101399 (2021). 10.1002/advs.202101399 PMC8425892 34231336

[54] Yu J (2013). Adaptive hydrophobic and hydrophilic interactions of mussel foot proteins with organic thin films. Proc. Natl Acad. Sci. USA..

