# Supplementary material for: Tough, stable and self-healing luminescent perovskite-polymer matrix applicable to all harsh aquatic environments
Source: Nat Commun. 2022 Mar 14;13:1338. doi: 10.1038/s41467-022-29084-z (PMC8921293; doi:10.1038/s41467-022-29084-z)
Supplement: Supplementary file 1 — Supplementary Information [file 41467_2022_29084_MOESM1_ESM.pdf]

Supplementary Information

Tough, stable and self-healing luminescent perovskite-polymer matrix applicable to all harsh aquatic environments

Yuncong Liu, [1,†] *Tao Chen,* [1,†] *Zhekai Jin,* [1] *Mengxue Li,* [1] *Dongdong Zhang,* [1] *Lian Duan,* [1] *Zhiguo Zhao*[2], *Chao Wang*[1,*]

[1]Key Lab of Organic Optoelectronics & Molecular Engineering, Department of Chemistry, Tsinghua University, Beijing 100084, PR China

[2]China Huaneng Clean Energy Research Institute, Beijing 102218, PR China

*Corresponding author. E-mail address: chaowangthu@mail.tsinghua.edu.cn
[†] These authors contributed equally.

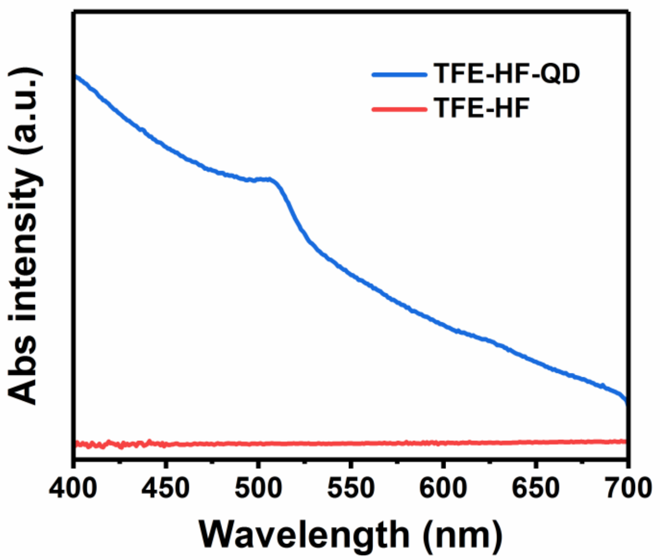

Supplementary Figure 1. UV-Vis absorption spectrum of TFE-HF$_{1.0}$ and TFE-HF-QD$_{1.0}$ film.

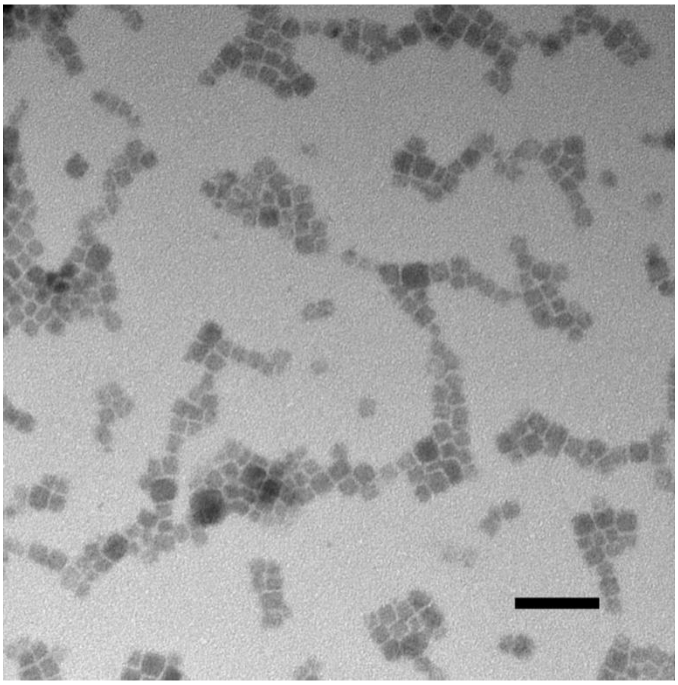

Supplementary Figure 2. TEM image of the synthesized $CsPbBr_3$. Scale bar, 50 nm.

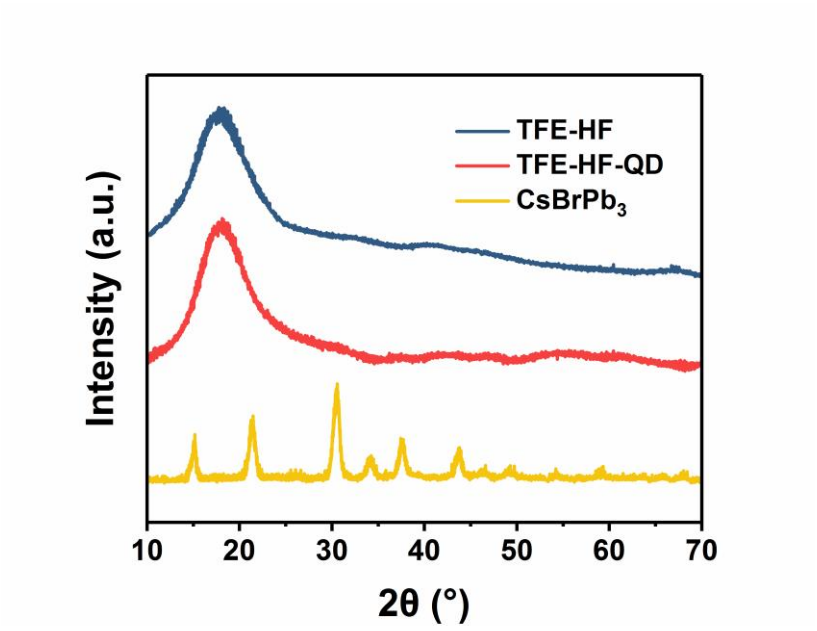

Supplementary Figure 3. The XRD patterns of $CsPbBr_3$, TFE-HF film and TFE-HF-QD film.

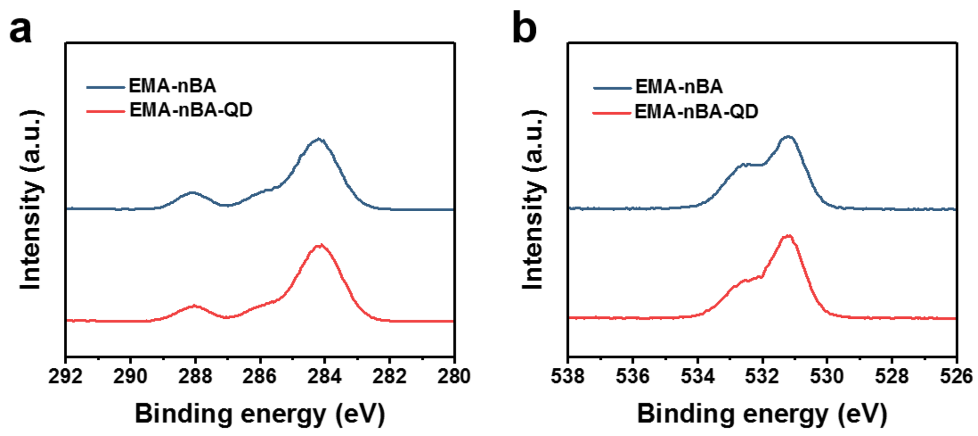

Supplementary Figure 4. XPS spectra of (a) C 1s and (b) O 1s region of EMA-nBA and EMA-nBA-QD.

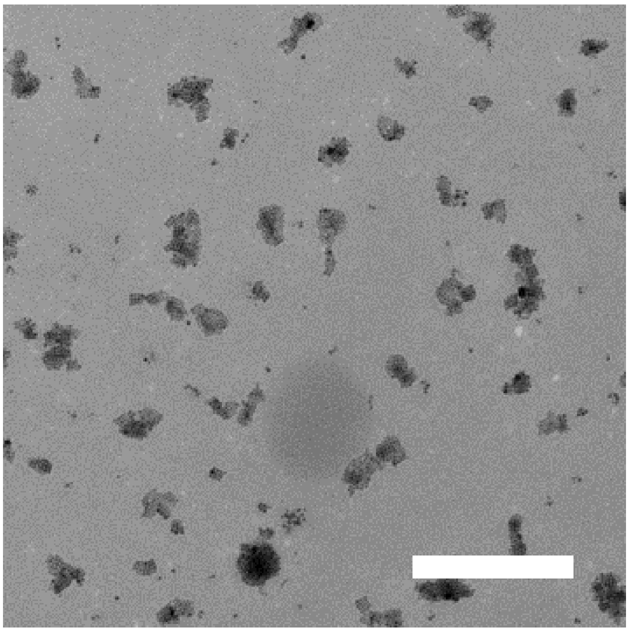

Supplementary Figure 5. A typical TEM image of EMA-nBA-QD. Scale bar, 0.5 μm.

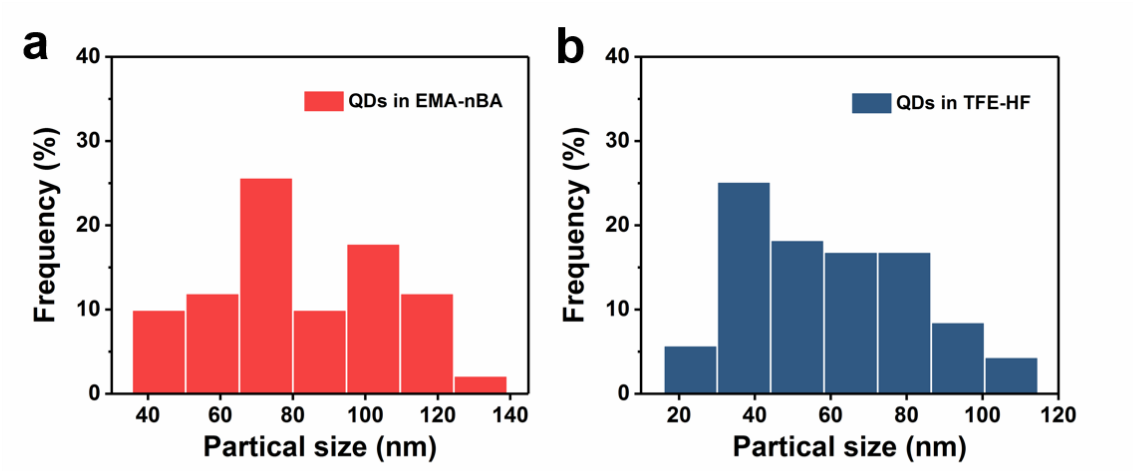

Supplementary Figure 6. Statistical diagram of particle size distribution in different polymers.

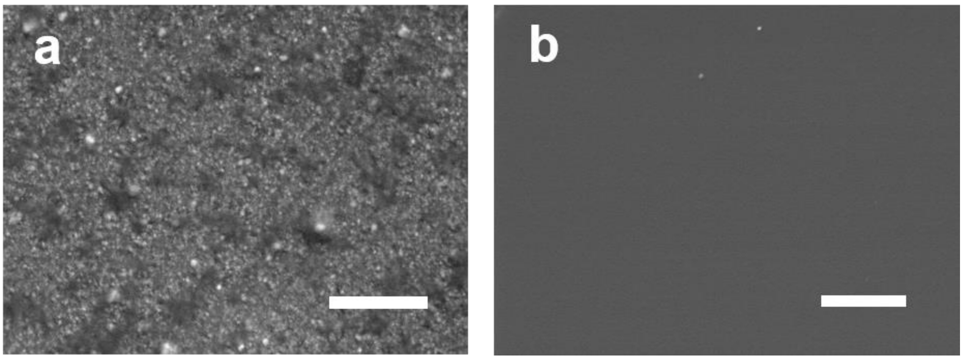

Supplementary Figure 7. SEM images of (a) $CsPbBr_3$ QDs and (b) TFE-HF-QD film. Scale bar, 50 μm.

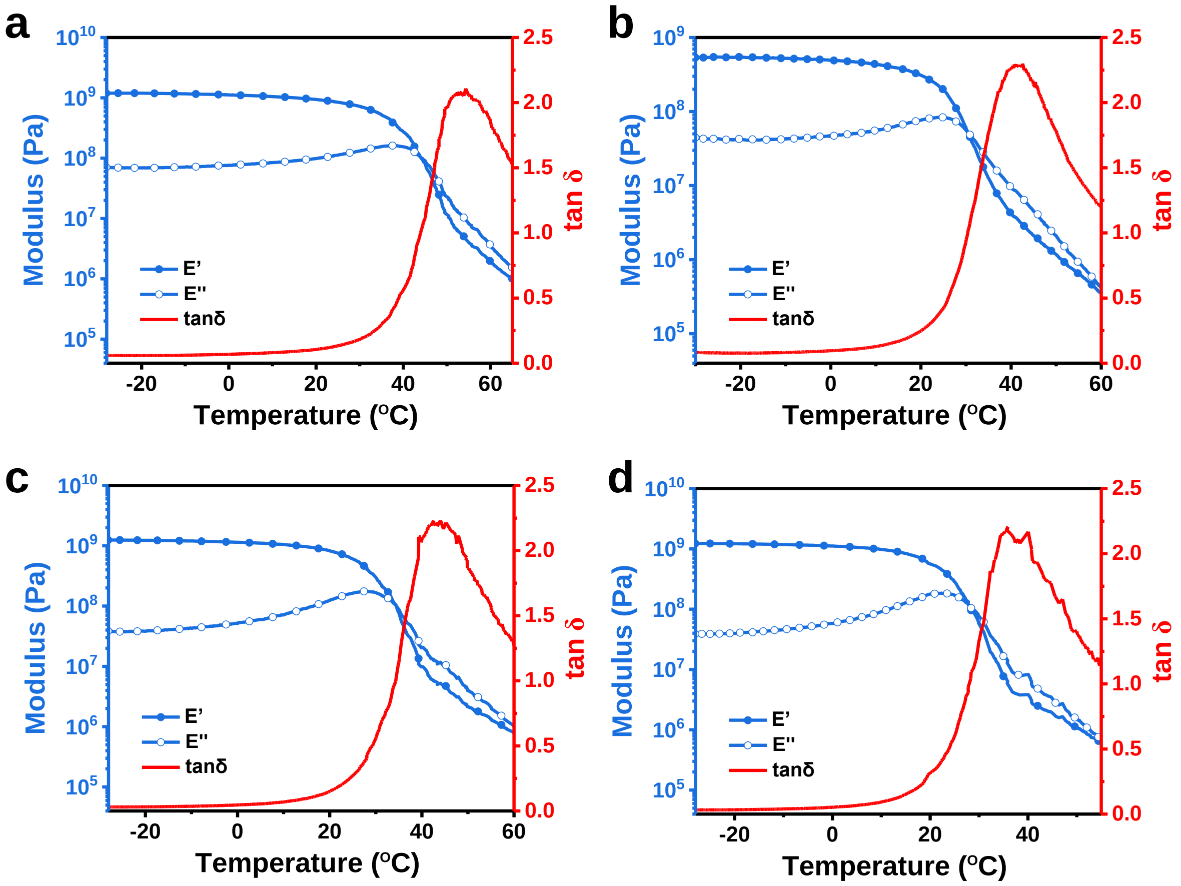

Supplementary Figure 8. DMA results. The storage modulus (G'), loss modulus (G'') and tan δ of (a) TFE-HF-$QD_{1.2}$; (b) TFE-HF-$QD_{1.0}$; (c) TFE-HF-$QD_{0.8}$ and (d) TFE-HF-$QD_{0.6}$.

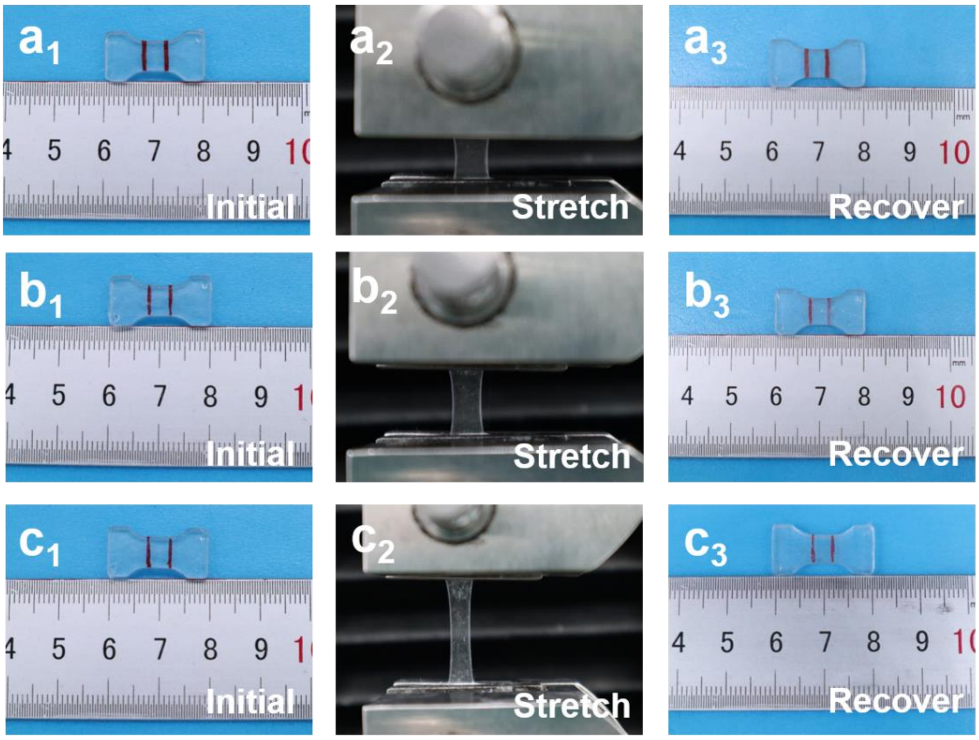

Supplementary Figure 9. Photos of stretching process. Photos of TFE-HF-$QD_{1.0}$ samples at initial state, stretched state and recovered after stretched to (a_1-a_3) 300%, (b_1-b_3) 500% and (c_1-c_3) 1000%.

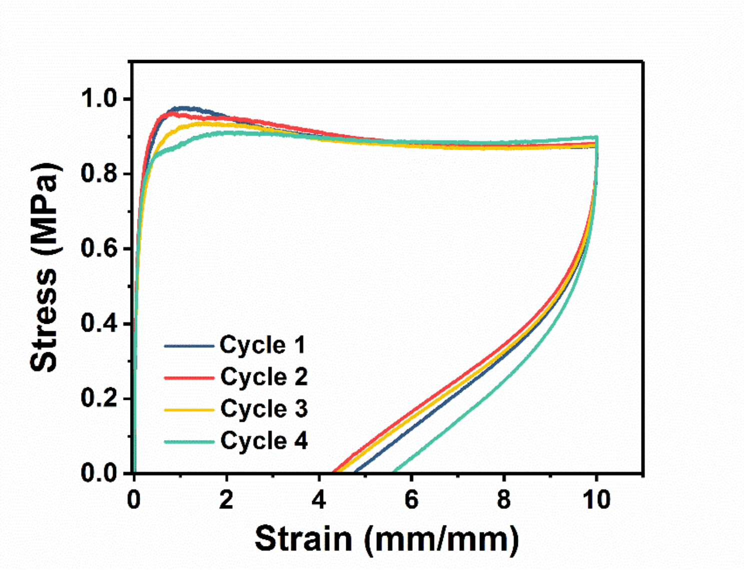

Supplementary Figure 10. Cyclic loading and recovery test. Cyclic loading and recovery of TFE-HF-$QD_{1.0}$ stretched to 10 times.

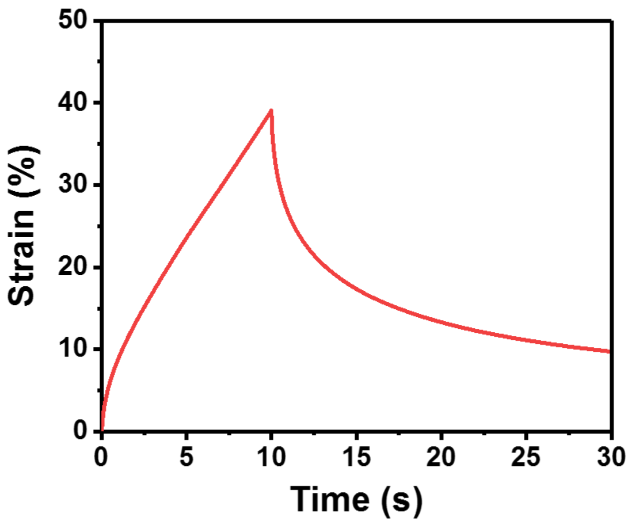

Supplementary Figure 11. Mechanical creep test of TFE-HF-QD$_{1.0}$. Rectangular samples (length: 16 mm; width: 6 mm; thickness: 2 mm) was used. Dead loads were used to apply constant engineering stresses of 0.3 MPa.

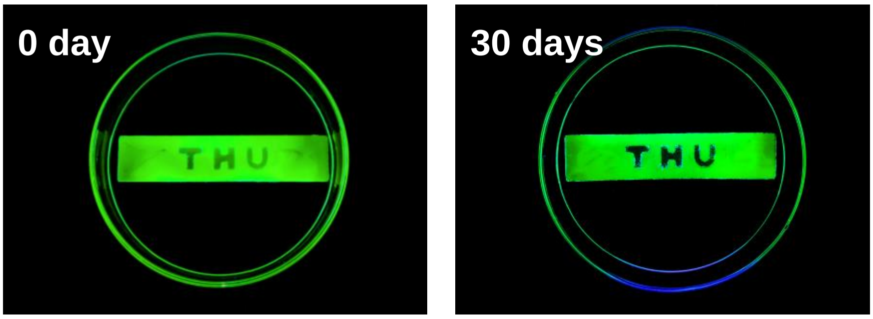

Supplementary Figure 12. Stability underwater. The photos of TFE-HF-$QD_{1.0}$ samples before and after immersed in water for a month.

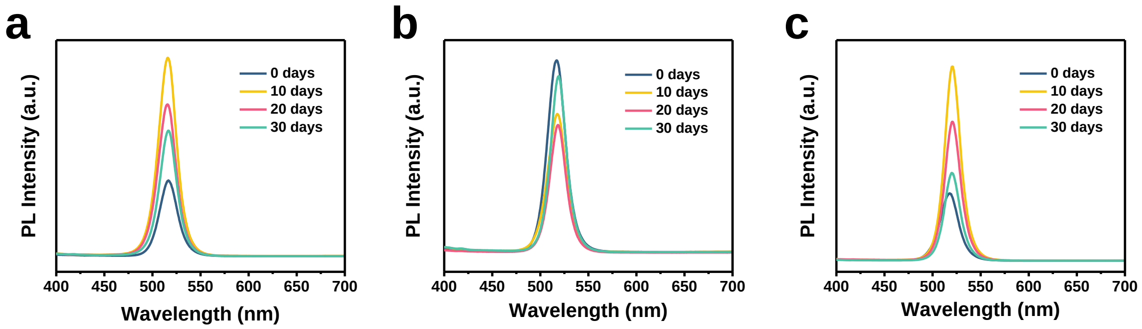

Supplementary Figure 13. PL intensity of TFE-HF-QD$_{1.0}$ after immersed in different aqueous environments. (a) In acid (pH=1); (b) In base (pH=13); (c) In salty water.

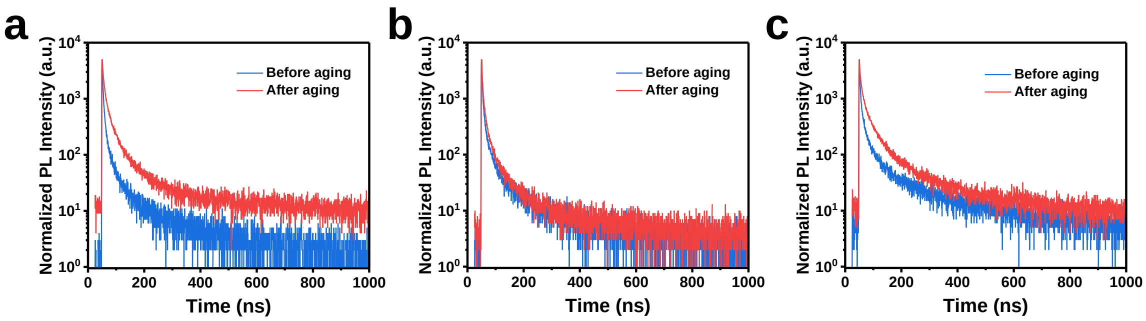

Supplementary Figure 14. PL decay dynamics of TFE-HF-QD$_{1.0}$ before and after aging in different conditions. (a) In acid solution (pH=1); (b) In base solution (pH=13); (c) In salty water.

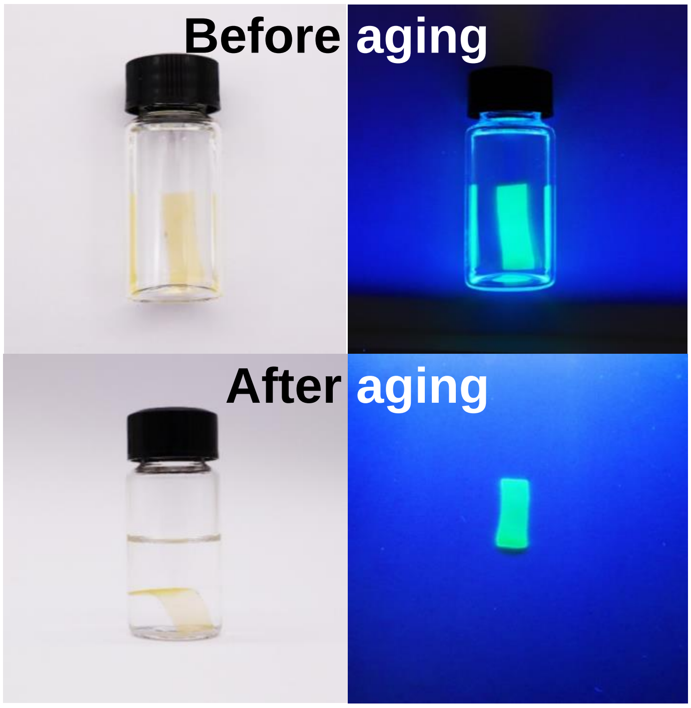

Supplementary Figure 15. The photos of TFE-HF-QD$_{1.0}$ samples before and after immersed in hexane for two weeks.

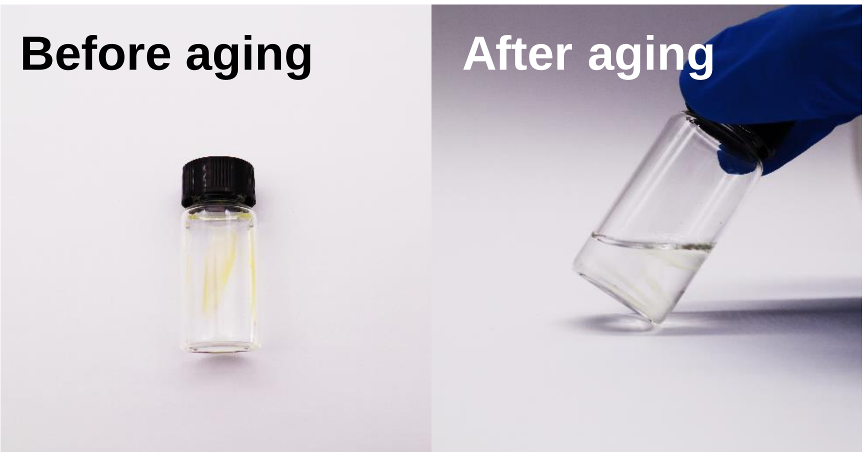

Supplementary Figure 16. The photos of SEBS-perovskite composite samples before and after immersed in hexane for two weeks.

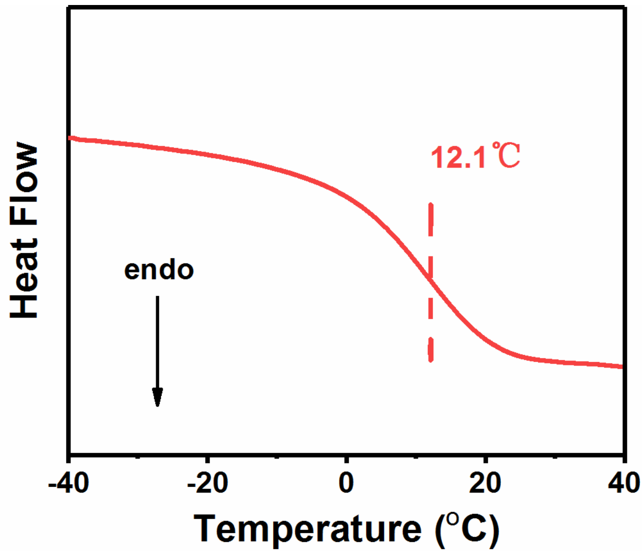

Supplementary Figure 17. DSC curve of TFE-HF-$QD_{1.0}$.

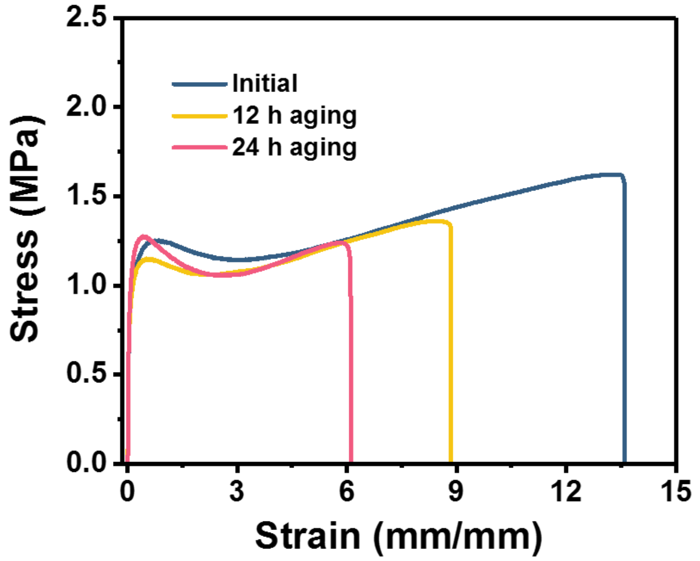

Supplementary Figure 18. Self-healing test for 12 h and 24 h aging samples.

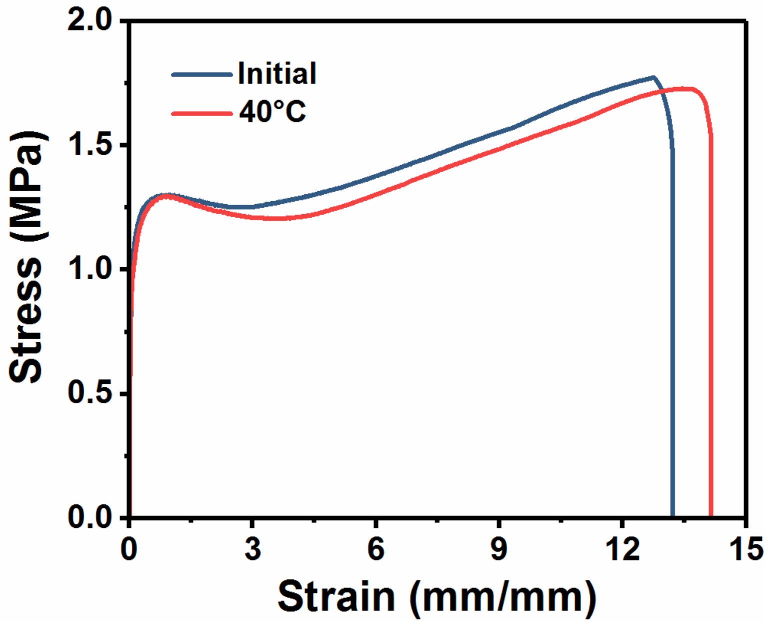

Supplementary Figure 19. Stress-strain curve of TFE-HF-QD$_{1.0}$ self-heal at 40°C for 24 h.

Supplementary Tables

Supplementary Table 1. Molecular weight information of different molar ratio polymers.

**Sample**	**M_n (Da)**	**M_w (Da)**	**PDI**
TFE-$HF_{1.2}$	49142	91904	1.870
TFE-$HF_{1.0}$	75100	131129	1.746
TFE-$HF_{0.8}$	61290	122794	2.003
TFE-$HF_{0.6}$	60450	74868	1.238

Supplementary Table 2. The absolute PLQY values. PLQY of TFE-HF-QD film after different aging process.

Condition	6 d	12 d	18 d	24 d	30 d
**TFE-HF-QD-Water**	49.92	29.53	27.42	26.27	24.16
**TFE-HF-QD-Acid**	39.07	28.25	29.80	26.71	22.74
**TFE-HF-QD-Base**	31.65	34.43	26.93	27.49	29.15
**TFE-HF-QD-Salty water**	44.77	41.49	30.57	30.03	33.30

Supplementary Table 3. Summary of PL lifetime before and after aging in different aqueous environments.

Conditions	τ_1 [ns]	τ_2 [ns]	τ_3 [ns]	Lifetime [ns]
Before aging	1.67	9.62	88.02	20.59
Aging in water	4.69	25.07	142.16	43.19
Aging in acid	5.09	24.25	118.85	40.17
Aging in base	2.77	15.88	111.87	31.87
Aging in salty water	4.95	29.86	134.45	54.06
