# Supplementary material for: Tough, stable and self-healing luminescent perovskite-polymer matrix applicable to all harsh aquatic environments
Source: Nat Commun. 2022 Mar 14;13:1338. doi: 10.1038/s41467-022-29084-z (PMC8921293; doi:10.1038/s41467-022-29084-z)
Supplement: Supplementary file 2 — Description of Additional Supplementary Files [file 41467_2022_29084_MOESM2_ESM.pdf]

Description of Additional Supplementary Files

File Name: Supplementary Movie 1
Description: Stretching process of TFE-HF-QD1.0 at a stretching speed of 40 mm min-1 under UV light.

File Name: Supplementary Movie 2
Description: A luminescent cotton fabric under deformation.

File Name: Supplementary Movie 3
Description: A luminescent cotton fabric in water environment.
